# Supplementary material for: Review of the Family Thanerocleridae (Coleoptera: Cleroidea) and the Description of Thanerosus gen. nov. from Cretaceous Amber Using Micro-CT Scanning
Source: Insects. 2022 May 6;13(5):438. doi: 10.3390/insects13050438 (PMC9147608; doi:10.3390/insects13050438)
Supplement: Supplementary file 1 [file insects-13-00438-s001.zip › Supplemental Table S1.pdf]

CHARACTER STATES MATRIX

	0 0 1 1 2 2 3
	1 5 0 5 0 5 0
Acanthocnemus	0 0 0 1 0 0 1 0 0 0 2 0 0 0 0 0 0 2 0 0 0 0 0 1 0 0 1 0 0 1 0 ? ?
Tenebroides	0 0 0 0 2 0 1 0 0 0 0 1 0 0 0 0 0 0 0 0 0 0 0 1 1 0 1 0 0 0 0 0 0
Clerus	0 0 1 1 2 0 0 1 1 1 0 0 0 0 0 1 1 0 1 0 0 0 0 0 0 1 0 0 0 1 1 0 1
Tilloidea	0 0 1 1 1 0 0 1 1 1 0 1 0 0 0 1 1 0 1 0 0 0 0 0 0 1 0 0 1 1 1 0 1
Archaeozenodosus	1 ? ? 1 2 0 0 ? ? 0 0 0 0 1 1 0 0 0 0 ? 0 0 0 ? ? ? ? ? ? 0 ? ? ?
Cretozenodosus	? ? 0 1 2 0 ? ? ? 0 0 0 0 1 1 0 0 0 0 ? 0 0 0 ? ? ? ? ? ? 0 ? ? ?
Mesozenodosus	1 ? ? 1 2 0 0 ? ? 0 0 0 0 ? 1 0 0 0 0 ? 0 0 0 ? ? ? ? ? ? 0 ? ? ?
Thanerosus	1 1 0 1 2 0 0 1 ? 0 1 0 0 0 1 0 0 0 0 1 0 0 0 ? ? ? ? ? ? 0 ? ? ?
Zenodosus	1 1 0 1 2 0 0 1 1 0 0 0 0 0 1 0 0 0 0 1 0 0 1 0 0 0 0 1 1 0 0 1 2
Meprinogenus	1 1 0 1 2 0 0 1 ? 0 0 1 1 1 1 0 0 0 0 1 1 1 1 1 1 0 0 1 1 1 1 ? ?
Neoclerus	1 1 0 1 2 1 0 1 ? 0 0 1 1 1 1 0 0 0 0 1 1 1 1 1 1 0 0 1 1 0 1 ? ?
Onerunka	1 1 0 1 2 0 0 1 ? 0 0 1 1 1 1 0 0 0 0 1 0 0 1 1 1 0 ? 1 1 0 1 ? ?
Thaneroclerus	1 1 0 1 2 0 0 1 0 0 0 1 1 1 1 0 0 0 0 1 1 1 1 0 0 0 0 1 1 0 1 1 2
Viticlerus	1 1 0 1 2 0 0 ? ? 0 0 1 1 1 1 0 0 0 0 1 1 1 1 – – 0 ? 1 1 1 1 ? ?
Ababa	1 1 0 0 2 0 0 1 ? 0 0 2 1 0 1 0 0 1 0 1 0 0 0 1 1 0 0 1 1 0 1 1 ?
Compactoclerus	1 1 0 0 2 1 0 2 1 0 1 1 1 1 1 0 0 1 0 1 0 0 0 1 1 0 0 1 1 0 1 ? ?
Isoclerus	1 1 0 1 2 0 0 2 1 0 1 1 1 1 1 0 0 1 0 1 0 0 0 1 1 0 0 1 1 0 1 1 ?
Parathaneroclerus	1 1 0 0 2 0 0 2 1 0 1 1 1 1 1 0 0 1 0 1 1 0 0 1 1 0 0 1 1 0 1 ? ?

CHARACTER LIST

1. Anterior edge of head: straight, without “horns” at sides = 0; medially emarginate, with frons with “horns” at sides = 1.

2. Gular sutures: narrowly separated at base, subparalel = 0; widely separated at base, convergent = 1.

3. Eye: not emarginate = 0; deeply emarginate anteriorly = 1.

4. Eye: not exceeding contour of head (flat) = 0; exceeding contour of head (elevate) = 1.

5. Antenna: non-clubbed = 0; serrate = 1; clubbed = 2.

6. Antenna: 11-segmented = 0; 10-segmented = 1.

7. Mandible: unidentate = 0; bidentate = 1.

8. Terminal palpomere of labial palps: coniform = 0; securiform = 1; truncate = 2.

9. Maxilla: lacinia simple = 0; lacinia divided (“lamina present”) = 1.

10. Prothorax, lateral carina: present = 0; absent = 1.

11. Depressions along notosternal suture: absent = 0; present = 1; present, with infrared receptor organ = 2.

12. Procoxal cavities externally: open = 0; closed = 1; imperfectly closed = 2.

13. Procoxa: transverse = 0; subspherical = 1.

14. Mesocoxal cavities: open = 0; closed = 1.

15. Protarsus: tarsomeres 1-4 moderate sized = 0; tarsomeres 1-4 shortened and widened, tarsus compact = 1.

16. Middle leg; lobes in tarsomeres 1-4: absent = 0; present = 1.

17. Tarsal claw: simple = 0; dentate = 1.

18. Tarsal pattern, male: 5-5-5 = 0; 5-4-4 = 1; 4-5-5 = 2.

19. Ultimate tarsomere as long as or longer than previous joints together: present = 0; absent = 1.

20. Base of femur prolonged to base of trochanter: absent = 0; present = 1.

21. Elytra, basal third: without depressions = 0; with depressions = 1.

22. Elytral gibbae: absent = 0; present = 1.

23. Elytral sculpture: regular = 0; irregular = 1.

24. Wing, 'wedge' cell: present = 0; absent = 1.

25. Radial cell: present = 0; absent = 1.

26. Aedeagus: inverted (ventrally open) = 0; uninverted (dorsally open) = 1.

27. Tegmen: in single part = 0; in three parts = 1.

28. Tegment, median strut: present = 0; absent = 1.

29. Tegment, lateral struts: present = 0; absent = 1.

30. Abdomen: five ventrites = 0; six ventrites = 1.

31. Abdominal segment IX: fully developed = 0; reduced to “spicular fork” = 1.

32. Larval urogomphi: hooked, present = 0; absent or strongly reduced in size = 1.

33. Larval mandible: prostheca multispinose = 0; bispinose = 1; prostheca absent or minute with single spine = 2.
